# Validation of the Subjective Spine Value: A new single item score for the assessment of spine-specific function

**DOI:** 10.1007/s00402-023-04941-8

**Published:** 2023-06-15

**Authors:** Vincent J. Leopold, Nikolas Warminski, Jannis Löchel, Kirsten Labbus, Matthias Pumberger, Robert K. Zahn

**Affiliations:** Department of Orthopaedic Surgery and Traumatology, Charité Berlin, University Hospital, Chariteplatz 1, 10117 Berlin, Germany

**Keywords:** Patient reported outcome measures, Subjective Spine Value, Spine disorders, Spinal function

## Abstract

**Background:**

Spinal function can be assessed through different patient reported outcome measures (PROMs). Purpose: The aim of the present study was to evaluate a novel single-item score for the assessment of spinal function: The Subjective Spine Value (SSpV). It was hypothesized that the SSpV correlates with the established scores Oswestry disability index (ODI) and Core Outcome Measures Index (COMI).

**Methods:**

Between 08/2020 and 11/2021 151 consecutive patients were prospectively enrolled and completed a questionnaire with the ODI, COMI as well as the SSpV. Patients were divided into 4 groups depending on their specific pathology (Group 1: Degenerative pathologies, Group 2: Tumor, Group 3: Inflammatory / Infection, Group 4: Trauma). Pearson correlation coefficient was used to evaluate correlation between the SSpV and the ODI and COMI separately. Floor and ceiling effects were evaluated.

**Results:**

Overall, the SSpV correlated significantly with both ODI (*p* =  < 0.001; *r* = − 0.640) and COMI (*p* =  < 0.001; *r* = − 0.640). This was also observed across all investigated groups (range − 0.420–0.736). No relevant floor or ceiling effects were noticed.

**Conclusions:**

The SSpV is a valid single-item score for the assessment of spinal function. The SSpV offers a useful tool to efficiently assess spinal function in a variety of spinal pathologies.

**Level of evidence:**

I, prospective cohort study.

## Background

Spinal disorders and injuries are among the most common pathologies of the musculoskeletal system and are associated with reduced health-specific quality of life [8, 25].

Patient Reported Outcome Measurements (PROMs) for the assessment of health-related quality of life and specific function are widely used tools in healthcare as well as in spinal surgery [2, 7, 11, 22]. They are essential to evaluate individual spine-specific function in patients with a variety of specific spinal disorders and their evaluation allows comparison of different forms of treatment and thus enables improvement of the quality of care [2]. A number of clinical scores for the evaluation of individual function are established in patients with spinal disorders [3, 5, 10]. However, the optimal set of outcome measures remains unknown [2, 4, 22].

Examples for such scores are the Oswestry Disablity Index (ODI) [6, 17] and Core Outcome Measures Index for the back (COMI-back) [15]. These multi-item scores are used in clinical practice to assess organ-specific function of the spine. As PROMs they are completed by the patient autonomously and are recorded independently of the physician.

However, such scores are often time-consuming to collect as well as to evaluate. In addition, complex questionnaires are prone to incomplete responses, which can limit their validity [19].

For this reason, subjective single item scores have been developed in musculoskeletal research for the (time-) efficient assessment of subjectively perceived joint-specific function [12, 13, 20, 26]. These valid scores show good correlation with multi-item scores and can be applied to a variety of pathologies and are regularly used in clinical practice. Furthermore, it has been shown that the response rate for single-item scores is higher than for more complex multi-item scores [20].

To our knowledge, no such single item score exists to date for assessing spinal function. Therefore, the aim of the present study was to validate a single-item score for the evaluation of individual spinal function: The Subjective Spine Value (SSpV).

## Methods

We conducted a prospective clinical trial and included patients who presented to our outpatient clinic for spine-specific complaints. Approval from the ethics committee was obtained beforehand (EA2/074/20). All patients signed informed consent.

### Inclusion criteria

Patients 18 years or older with a specific diagnosis for which they presented to our outpatient clinic at a university hospital were included.

### Exclusion criteria

Patients younger than 18 years or who had a language barrier that compromised their ability to answer the questionnaire were excluded from participation.

### Patient recruitment & data collection

Between 08/2020 and 11/2021 a total of 151 consecutive patients presenting to our outpatient clinic for specific spinal pathologies were prospectively enrolled. Patients were given a spine-specific questionnaire and completed it independently before physician consultation. In addition to basic demographic data, the questionnaire included the SSpV as well as the ODI [5] and COMI [15]. It was hypothesized that the SSpV significantly correlates with the established scores Oswestry disability index (ODI) and Core Outcome Measures Index (COMI).

The ODI is one of the most commonly used PROMs in spinal disorders and is applied in surgically as well as non-surgically treated patients. In 10 sections it assesses the level of pain and interference with physical activities, sleeping, personal care, social life, sex life, and traveling. Each item is scored from 0 to 5 (0, indicating little disability, to 5, indicating severe disability) resulting in score between 0 and 100, with a higher score indicating higher disability as perceived by the patient [6, 17].

The COMI-back is also a widely used PROM in spinal disorders as it has been incorporated into the Eurospine Spine Tango registry as their outcome measure of choice [18, 21, 27]. It is a multidimensional instrument that covers the domains pain (back and leg/buttock pain intensity, each measured separately on a 0–10 numeric graphic rating scale), back-related function, symptom-specific well-being, general quality of life, social disability and work disability (each scored on a 5-point scale). The COMI-back score ranges from 0 to 10 with a higher score indicating higher disability [14, 16].

To record the SSpV, all patients were asked to grade the individual function of their spine. The maximum score is 100% which is equivalent to no complaints and problem-free spine function. The minimum score is 0% indicating a severe spinal problem. In reference to previously published single-item scores such as the Subjective Shoulder Value (SSV), Subjective Hip Value (SHV), or Subjective Knee Value (SKV), patients were asked to answer the following question, "What percentage is the function of your spine if the normal function of a healthy spine is 100%? (or: How many euros is your spine worth if a normal spine is worth 100 euros?)" [12, 13, 20].

Based on a synopsis of anamnesis as well as clinical and radiological examination, the specific diagnosis was made by a senior spine surgeon (RZ).

Patients were divided into 4 groups based on the specific spinal pathologies: Group 1: Degenerative pathologies (*n* = 65), Group 2: Tumors (*n* = 26), Group 3: Inflammation/Infect (*n* = 7), Group 4: Trauma (*n* = 53). This clustering was based on the registry of the German Spine Society.

### Statistics

Statistical analysis was performed using SPSS Statistics 25.0 software (IBM, Armonk, NY, USA). P values less than 0.05 were considered statistically significant. Descriptive statistics (Frequency rates, means, and range, floor/ ceiling rates) were utilized to describe baseline patient characteristics. Construct validity requires that different measures of a similar or the same construct agree to an acceptable extent [1, 24]. In the present study construct agreement was evaluated using Pearson correlation coefficients. Depending on the correlation coefficient r, the correlations were interpreted as excellent (0.81–1.00); very good (0.61–0.80); good (0.41–0.60); fair (0.21–0.40); and poor (0.00–0.20) [9]. It was hypothesized that correlation coefficients would range from 0.4 to 0.8 when each score measured similar underlying attributes [23].

Since ODI and COMI are inversely scored compared to the SSpV, negative *r* values are calculated in the correlation analysis. A high value indicates a higher degree of disability in ODI and COMI, while in the SSpV a higher value indicates better spinal function.

## Results

A total of consecutive 151 complete datasets were analyzed. Of the included patients 70 (46%) were female and 81 (54%) were male. Mean age was 62 years (SD 16; range 20–88). For a detailed summary of baseline demographics and PROMs, see Table 1. Floor and ceiling effects were comparable across all PROMs (SSpV: 2–2%; ODI: 0–1%; COMI: 1–2%).

**Table 1:** Baseline demographic data and PROMs for the entire study population and by group (Group 1 = Degenerative pathologies, Group 2 = Tumor, Group 3 = Inflammation/Infect, Group 4 = Trauma

	*N* =	Age	SSpV	ODI	COMI
Overall	151	62 ± 17	52 ± 26	44 ± 94	6 ± 2.7
Group 1	65	60 ± 17	50 ± 27	44 ± 21	6 ± 2.6
Group 2	26	67 ± 9	45 ± 24	50 ± 23	7 ± 2.4
Group 3	7	62 ± 18	55 ± 33	48 ± 27	5 ± 3.7
Group 4	53	61 ± 20	57 ± 30	42 ± 25	5 ± 2.6

For the overall study population, the SSpV correlated significantly with the ODI (*r* = − 0.640; *p* =  < 0.001) and the COMI (*r* =− 0.640; *p* =  < 0.001).

Among the different subgroups, a good to very good correlation was found among all 4 groups ranging from − 0.420 to − 0.736.

**Fig. 1:**
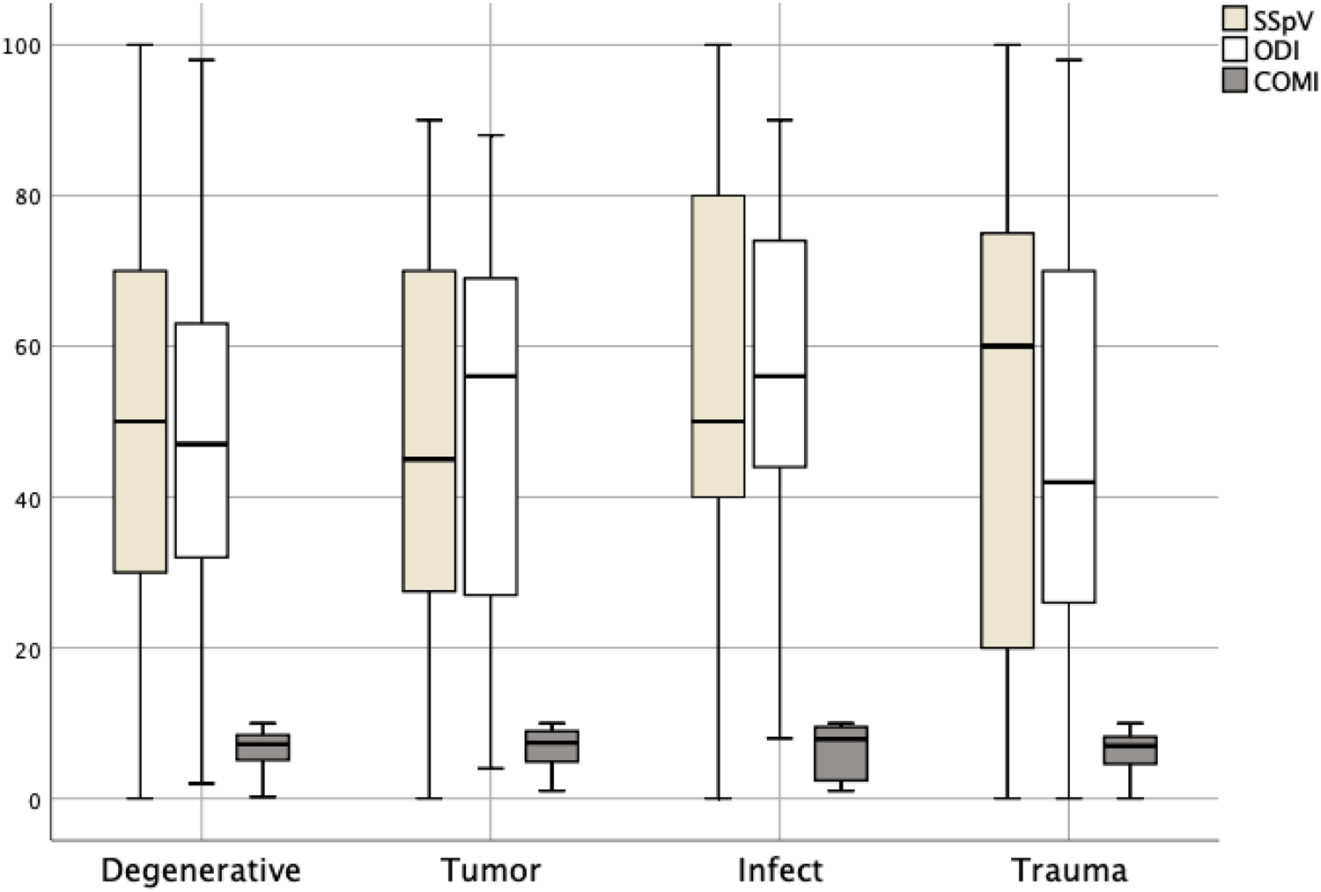
Patient reported outcome measurements for the different groups. Consider the different scale (Left to right: SSpV, ODI, COMI). The boxplots illustrate median, lower and upper quartile. Whiskers represent lowest and highest scored values

Correlations between ODI and COMI were also significant for the total population (*r* = 0.692; *p* =  < 0.001) as well as for the subgroups. In the subgroups, the correlations ranged between 0.574 and 0.793 (see Fig. 1).

For a detailed description of the individual correlations, see Table 2.

**Table 2:** Correlations between SSpV and ODI as well as COMI; Interpreted as excellent (0.81–1.00); very good (0.61–0.80); good (0.41–0.60); fair (0.21–0.40); and poor (0.00–0.20) [9]

	*r*	*p* value	Relationship
Overall (*n* = 151)			
SSpV vs. ODI	– 0.640	< 0.001	Very good
SSpV vs. COMI	– 0.640	< 0.001	Very good
ODI vs. COMI	0.692	< 0.001	Very good
Group 1 (*n* = 65)			
SSpV vs. ODI	– 0.710	< 0.001	Very good
SSpV vs. COMI	– 0.736	< 0.001	Very good
ODI vs. COMI	0.793	< 0.001	Very good
Group 2 (*n* = 26)			
SSpV vs. ODI	– 0.517	0.001	Good
SSpV vs. COMI	– 0.420	0.011	Good
ODI vs. COMI	0.719	< 0.001	Very good
Group 3 (*n* = 7)			
SSpV vs. ODI	– 0.710	0.001	Very good
SSpV vs. COMI	– 0.738	0.001	Very good
ODI vs. COMI	0.706	0.098	Very good
Group 4 (*n* = 53)			
SSpV vs. ODI	– 0.611	< 0.001	Very good
SSpV vs. COMI	– 0.551	< 0.001	Good
ODI vs. COMI	0.574	< 0.001	Good

## Discussion

The most important finding of the presented prospective study is that the SSpV shows high and statistically significant correlation with the established PROMs ODI and COMI for the assessment of spinal function. Significant correlations were found in the overall study population as well as within the 4 subgroups. It can therefore be assumed that the SSpV measures the same underlying attributes of spinal function as the ODI as well as the COMI [23].

The ODI is considered the gold standard among PROMs in spinal disorders [6]. Completion of the questionnaire takes 3.5–5 min and scoring approximately 1 min [5] making its collection time-consuming in everyday clinical practice. It has been shown that more complex multi-item PROMs are more prone to errors in evaluation. In a study by Mehra et al. it was shown in an evaluation of the ODI that up to 33% of the questionnaires were not scored correctly [17]. More complex and time-consuming scoring may also result in no PROMs being collected at all. A survey of AO Spine members worldwide showed that 31.9% of respondents do not routinely collect PROMs at all. The main reasons cited for not collecting PROMs were a lack of time to collect the questionnaires, as well as a lack of staff to assist with data collection and the long time to fill out the questionnaires [7]

These problems can be addressed with validating the SSpV as a single-item score, as it can be collected intuitively and time-efficiently by the patient and interpreted by the physician at a glance.

Faster to answer and more intuitive single-item scores can also lead to higher response rates and more fully completed questionnaires, as Plachel et al. showed with a similar score for the knee. Here, significantly higher rates of complete responses were shown for the single-item score compared to more complex multi-item scores [20].

In previous studies validating single-item values, higher correlations with established scores were reported for the Subjective Knee Value (SKV) or the Subjective Hip Value (SHV) [13, 20].

This may be in part due to the fact that the spine itself is a more complex anatomical construct within the musculoskeletal system than the large joints of the extremities and presents with more diverse clinical symptoms with pain sometimes extending beyond the spine.

Interestingly, in the work presented, similar correlations were found between SSpV and ODI/COMI as between ODI and COMI themselves.

This is consistent with the literature in so far as similar values are regularly found in correlations of established scores measuring spinal function. This could suggest that spinal function is more difficult to measure overall, or that measurements need to be more specific to individual spinal pathologies [21].

The study presented must be viewed in the light of its imitations. First the patient population studied does not represent the entire spectrum of spinal pathologies. Further studies are needed to investigate the SSpV in other pathologies such as spinal deformities. However, a variety of different pathologies were represented in the study cohort.

Second, is the relatively high mean age in the investigated study population of 62 years. Future studies could investigate the influence of age on PROMs and evaluate the SSpV in a younger population.

Third, while validity was tested in the presented study, other psychometric properties (i.e. reliability, responsiveness) were not tested. In further studies, the SSpV needs to be investigated as a follow-up parameter and whether it responds to changes after therapy.

## Conclusion

This is the first study to report a valid single-item measure for the assessment of spinal function. The SSpV shows high correlation with established spine specific outcome scores in a patient population with a variety of spine-specific disorders.

It thus offers a useful tool for assessing spinal function, as it is quick and easy to collect and interpret.
